# The Beneficial Effects of Resveratrol on Hepatocellular Carcinoma and Nonalcoholic Fatty Liver Disease: Modulation of Apoptosis, Autophagy, Inflammation, and Oxidative Stress

**DOI:** 10.1002/fsn3.70555

**Published:** 2025-07-24

**Authors:** Ziyao Wan, Jamal Hallajzadeh

**Affiliations:** ^1^ School of Pharmacy University College London London UK; ^2^ Research Center for Evidence‐Based Health Management Maragheh University of Medical Sciences Maragheh Iran

**Keywords:** apoptosis, autophagy, HCC, NAFLD, resveratrol

## Abstract

Nonalcoholic fatty liver disease (NAFLD) is characterized by excessive liver fat accumulation that can progress to the more severe NASH. Impaired autophagy, the cellular process of recycling damaged organelles and proteins, has been observed in NAFLD. Interestingly, the compound resveratrol has been shown to induce autophagy in liver cells, potentially helping to alleviate NAFLD. Resveratrol can also trigger apoptosis, or programmed cell death, in hepatocellular carcinoma (HCC) cells, the most common form of liver cancer, by inhibiting key signaling pathways. This apoptosis‐inducing effect highlights resveratrol's therapeutic potential for HCC. Additionally, the cytotoxicity of resveratrol in cancer cells suggests that it may selectively target and eliminate damaged or malignant cells, further contributing to its anticancer properties. Resveratrol activates the AMPK protein complex, which regulates energy balance and autophagy, suggesting it may help remove damaged cellular components in NAFLD. While preclinical studies are promising, further clinical research is still needed to evaluate the efficacy and safety of resveratrol for treating NAFLD and HCC.

## Graphical abstract

Resveratrol protects against NAFLD and HCC by modulating apoptosis, autophagy, oxidative stress, and inflammation. Resveratrol activates key signaling pathways like PI3K/AKT/mTOR and SIRT1 to promote autophagy and reduce pro‐apoptotic and inflammatory responses. In advanced HCC, resveratrol's anti‐apoptotic effects may support tumor growth, emphasizing the need to tailor its use to disease stage and context.

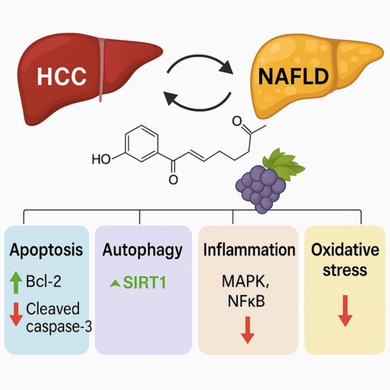

## 1 Introduction

Apoptosis, another name for programmed cell death, is an essential process in the onset, spread, and management of hepatocellular carcinoma (HCC) (Kouroumalis et al. 2023). A characteristic of HCC is dysregulation of apoptosis, which leads to tumor development, treatment resistance, and worse patient outcomes. Therefore, one possible treatment approach for HCC is to target the mechanisms involved in apoptosis. HCC is a significant global health issue as it is the leading cause of a significant percentage of cancer‐related deaths worldwide. A viable strategy for enhancing treatment results is comprehending the dysregulation of apoptotic pathways in HCC and creating tailored therapeutics to restore apoptosis. In order to preserve and enhance cellular viability, autophagy is a biological process that entails the breakdown and recycling of many cellular constituents. The name “autophagy” refers to this act of eating itself; it is derived from the Greek words “auto” (self) and “phagy” (to eat). Nonalcoholic fatty liver disease (NAFLD) is a disorder marked by the buildup of fat in the liver without the presence of excessive alcohol use. Autophagy is important in the development and progression of NAFLD (Pourteymour et al. 2023). Autophagy is an essential process that keeps the liver homeostatic by clearing hepatocytes of excess lipid droplets and damaged organelles. It serves as a safeguard against the onset and advancement of NAFLD. People with NAFLD have been shown to have impaired autophagy, and this impairment may play a part in the illness's development (Ren et al. 2024).

A natural polyphenol in many plants, resveratrol, has been investigated for possible therapeutic benefits for NAFLD, including the induction of death in liver cells. Studies have demonstrated that resveratrol induces apoptosis in hepatocytes. Resveratrol can induce apoptosis through various pathways, including death receptor‐mediated and mitochondrial mechanisms (Jang, Im, and Kim 2022). By causing cytotoxicity and apoptosis in hepatocytes, resveratrol may help remove damaged or malfunctioning cells and lessen liver injury by causing apoptosis in hepatocytes. Resveratrol has been discovered to reduce hepatic steatosis, or the buildup of fat in the liver, in both animal and cell culture models of NAFLD. By eliminating too many hepatocytes packed with fat, resveratrol's activation of apoptosis in liver cells can help lower hepatic steatosis (Schwabe and Luedde 2018). Resveratrol has anti‐inflammatory properties. It can inhibit the liver's synthesis of chemicals that promote inflammation. Resveratrol may help alleviate the progression of NAFLD by reducing inflammation (Meng et al. 2021). One important aspect of the development of NAFLD is chronic inflammation (Schwabe and Luedde 2018). Resveratrol may indirectly encourage apoptosis and impede the development of NAFLD by lowering inflammation. Due to its antioxidant properties and ability to scavenge free radicals, resveratrol helps protect the liver from oxidative stress. A later stage of NAFLD characterized as liver fibrosis and hepatocyte death are both known to be influenced by oxidative stress. Further evidence for resveratrol's ability to prevent or treat liver damage associated with NAFLD comes from its antioxidant and anti‐fibrotic properties.

## 2 Resveratrol

### 2.1 General Properties of Resveratrol

**FIGURE 1:**
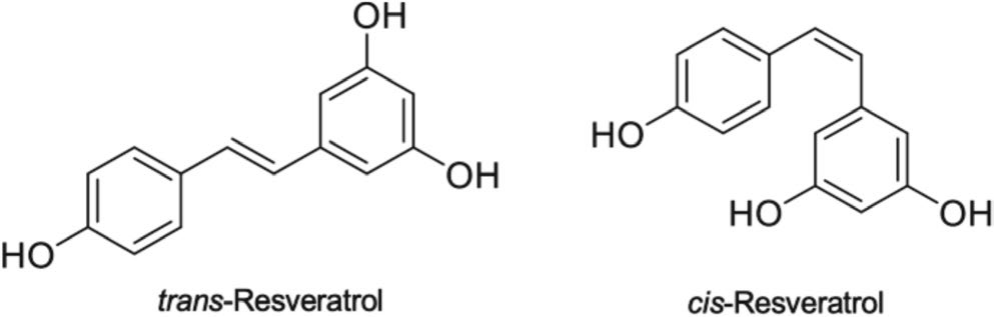
The chemical structure of resveratrol.

Resveratrol, or 3,5,4′‐trihydroxystilbene, is a phytoalexin that some plants make to protect themselves against the damaging effects of fungi (Chen et al. 2001; Shabani Nashtaei et al. 2017). Resveratrol is synthesized by the enzyme resveratrol synthase, which utilizes the Shikimate pathway to combine three molecules of malonyl‐coenzyme A with p‐coumaroyl‐coenzyme A. Plant defense chemicals and elicitors control the activity of the enzyme (Medina‐Bolivar et al. 2007). There are two different isomeric forms of resveratrol: cis and trans (Figure 1). The trans form is more stable and has higher bioactivity than the cis form (Prokop et al. 2006). On the other hand, the trans form can change into the cis form when exposed to UV radiation. The action of resveratrol in its glucose‐bound glycosylated form differs from that of its aglycan form. The action of glucosidase in the human digestive system can be influenced by its presence (Chen et al. 2001; Prokop et al. 2006). Alcoholic extracts can be used to extract resveratrol effectively (Cho et al. 2006). Grapes and grape products, especially grape skins and seeds, are the richest natural dietary source of resveratrol. Resveratrol is a hormone present in plants. Its levels can fluctuate wildly; as grapes grow, biotic stressors such as fungal infections or UV radiation cause them to display larger amounts. Higher amounts are found in grapes cultivated in colder areas. However, compared to red wines and grape juice, chocolate and other cocoa items have less trans‐resveratrol (Hurst et al. 2008).

Additionally, resveratrol has been studied for its cytotoxicity against cancer cells, particularly in HCC. Its ability to induce apoptosis in cancer cells contributes to this cytotoxic effect, making it a potential therapeutic agent. Resveratrol oligomers show a variety of biological actions and are primarily present in particular plant groups (Ito et al. 2009).

### 2.2 Bioavailability

Previous research has examined the rates of resveratrol absorption in laboratory and animal studies (Walle 2011; Gowd et al. 2019). Numerous studies have indicated that resveratrol is well‐absorbed following oral intake (Frémont 2000). However, the concentration of resveratrol in the bloodstream typically remains relatively low in the nanomolar range (Gowd et al. 2019). Moreover, due to its intricate structure and substantial molecular weight, the efficient metabolism of resveratrol in the liver and intestine results in an oral bioavailability of approximately 12% for trans‐resveratrol (Walle 2011). Resveratrol is taken up into the enterocytes of the intestinal lining through passive diffusion or carrier‐mediated transport across the apical membrane. Subsequently, it undergoes rapid and extensive metabolism, primarily through conjugation with glucuronic acid or sulfate. This metabolic process results in resveratrol glucuronides or sulfates (Kuhnle et al. 2000; Yu et al. 2002; Lançon et al. 2004).

In contrast, a substantial proportion (around 90%) of ingested resveratrol remains unchanged as it reaches the colon, where it undergoes fermentation by the gut microbiota. After being absorbed through the portal vein, the polyphenolic metabolites generated from resveratrol undergo additional modifications in the liver, such as methylation, glucuronidation, or sulfation. These modified metabolites then enter the systemic circulation and travel to target tissues and cells, where their physiological effects become apparent. Additionally, the limited impact on chemopreventive parameters, such as phase I and phase II enzymes, total antioxidant activity, and vitamin E status, was attributed to resveratrol conjugates' low or absent activity (Corder et al. 2003). Resveratrol and any unutilized metabolites can be recycled back to the small intestine through bile or excreted from the body through urine (Gowd et al. 2019). The ABC membrane proteins expel the previously stated metabolites; however, resveratrol is not. Consequently, resveratrol's metabolism in intestinal cells likely causes its poor bioavailability. Acetylated hydroxyl groups have a superior pharmacokinetic profile and greater stability than hydroxyl groups because they do not undergo glucuronidation, whereas hydroxyl groups undergo sulfation and glucuronidation more often (Liang et al. 2013).

Additionally, due to its increased hydrophobicity, acetylated resveratrol more deeply penetrates the cellular membrane than the parent molecule (Lançon et al. 2004). According to a recent study, trans‐resveratrol metabolites are secreted into the intestinal lumen via ABC transporters. Transporters that function as a “bottleneck” in the absorption of trans‐resveratrol are probably responsible for the distribution and bioavailability of resveratrol metabolites in different organs (Planas et al. 2012). It was discovered in the kidney, liver, lung, spleen, stomach, small intestine, and brain after resveratrol administration, among other organs. The lung and heart were where the chemical was most commonly found (Liang et al. 2013).

## 3 Pathophysiological Mechanisms of Progressive NAFLD

Various genetic predispositions combine with intra‐ and extrahepatic processes, such as altered gut microbiota and dysfunctional adipose tissue, to cause the development of NAFLD (Friedman et al. 2018; Pafili and Roden 2021; Buzzetti et al. 2016; Lebeaupin et al. 2018). The primary cause of NAFLD is hepatocellular lipid buildup, which develops when the liver's ability to handle fats and carbohydrates is surpassed. Accumulated hazardous lipid species induce lipotoxicity through interactions with several cellular pathways. Lipotoxicity is likely the cause of the establishment of insulin resistance and cellular stress, damage, and ultimately death (Friedman et al. 2018). However, the body has a defense mechanism in place. Toxic lipid species can trigger an adaptive stress response called the unfolded protein response (UPR), which protects cells and induces endoplasmic reticulum (ER) stress to restore cellular homeostasis. Chronic UPR activation in NAFLD may exacerbate insulin resistance and lipid buildup. It may also accelerate the development of NASH by causing oxidative stress, inflammation, and hepatocellular death (Lebeaupin et al. 2018). NASH has also been linked to decreased hepatocyte oxidative capability and mitochondrial dysfunction. This increases the production of reactive oxygen species (ROS), which worsens lipotoxicity and leads to oxidative stress, inflammation, and fibrosis (Buzzetti et al. 2016). Kupffer cells, macrophages found in the liver, are activated during the development of NASH and release chemokines and cytokines, respectively (Tacke 2017). This leads to changes in the immunological environment of the liver, including the invasion of immune cells like neutrophils and monocytes, and it also exacerbates the chronic inflammatory condition of the liver (Huby and Gautier 2022).

Additionally, it has been demonstrated that liver‐resident macrophages, regardless of their inflammatory state, contribute to insulin resistance by producing non‐inflammatory molecules that directly control hepatocyte insulin signaling and liver metabolism (Morgantini et al. 2019). Hepatic stellate cells (HSCs) are stimulated by stimuli derived from both liver‐resident macrophages and stressed hepatocytes to produce myofibroblasts and deposit exogenous amounts of extracellular matrix (ECM) proteins (Higashi et al. 2017). Changes in miRNA expression are just one aspect of the deep gene expression reprogramming that underlies NAFLD. The role of miRNA in this reprogramming is significant, and its potential in understanding and treating NAFLD is vast. Since the first report on changes in hepatic miRNA expression patterns in human NASH (Cheung et al. 2008), researchers have concentrated on miRNAs as potential contributors to the development and course of this more prevalent illness. These studies aim to understand the molecular processes reprogramming gene expression in NAFLD.

## 4 Role of Resveratrol in NAFLD: Focus on Inflammation and Oxidative Stress

Countless studies have delved into the potential benefits of resveratrol for NAFLD. Resveratrol has been found to alter numerous cellular and molecular pathways in the context of NAFLD, offering a ray of hope for improving liver health. By upregulating the expression of the scavenger receptor class B type I (SRB1) and low‐density lipoprotein receptor (LDLR) genes in the liver, resveratrol executes its protective actions (Xin et al. 2013). These receptors play a crucial role in lipid metabolism and cholesterol removal from circulation. The increased expression of these receptors by resveratrol promotes the uptake and clearance of lipids, thereby reducing fat accumulation in the liver. Resveratrol has also been shown to regulate autophagy, a cellular mechanism that breaks down and recycles faulty or surplus cellular components (Li et al. 2014). This aids in maintaining cellular homeostasis and preventing lipid buildup in the liver.

Moreover, resveratrol has been shown to inhibit the activity of NF‐κB, a transcription factor involved in immunological responses and inflammation (Li et al. 2014). This effect is achieved by restoring the nuclear factor of kappa light polypeptide gene enhancer in B‐cells inhibitor α (IκBα). Resveratrol alleviates oxidative stress and inflammation in the liver, two prominent symptoms of NAFLD, by inhibiting NF‐κB activation (Li et al. 2014). This anti‐inflammatory property of resveratrol provides a reassuring prospect for its potential in NAFLD treatment.

Furthermore, resveratrol has been reported to enhance the numbers of CD68+ Kupffer cells, which are specialized immune cells in the liver responsible for the clearance of pathogens and cellular debris (Nishikawa et al. 2015). By promoting the activity of these cells, resveratrol aids in removing accumulated lipids and inflammatory molecules from the liver, contributing to the improvement of fatty liver. In addition to its effects on autophagy and apoptosis, resveratrol has also been shown to inhibit the expression of proteins involved in the differentiation and accumulation of adipose (fat) cells. By downregulating these pro‐adipogenic factors, resveratrol can help prevent excessive fat deposition within the liver, thereby aiding in the prevention and management of NAFLD. This suppression of adipocyte differentiation and fat cell buildup represents another important mechanism by which resveratrol exerts its protective effects against the development of fatty liver disease (Nishikawa et al. 2015). High‐content atherogenic diets produce substantial liver damage in both humans and experimental animals, which is the cause of fatty liver disease. Research using a high‐fat diet (HFD) model of liver damage has demonstrated the hepatoprotective effects of resveratrol. The aforementioned research highlights the potential advantages of resveratrol in lowering cholesterol, triglyceride, and lipid levels, in addition to its effects on lipid metabolism‐related gene expression and enzyme function. In research done on mice, Ahn et al. (2008) supplemented the diet with resveratrol. The mice's cholesterol, triglycerides, and lipid levels were shown to decrease dramatically with resveratrol supplements.

Furthermore, it suppressed the expression of genes linked to lipid metabolism that a high‐fat meal had enhanced. From this, resveratrol could offer some protection against dyslipidemia, a condition characterized by abnormal levels of lipids in the blood, brought on by diet. Resveratrol was given to rats on an HFD for 8 weeks in a different trial conducted by the same investigators (Ahn et al. 2007). The researchers discovered that resveratrol stopped the production of the inflammatory enzyme nitric oxide synthase, decreased levels of TNF‐α, a sign of inflammation, and stopped lipid peroxidation. Additionally, the enzymes catalase (CAT), superoxide dismutase (SOD), and glutathione peroxidase (GPx) all had increased activity under resveratrol (Ahn et al. 2007). The significant functions of these enzymes in antioxidant defense imply that, when combined with an HFD, resveratrol may have anti‐inflammatory and antioxidant properties. Rats on an HFD showed reduced cholesterol and lipid levels when resveratrol was added to the same experimental setup for a second study.

Furthermore, there was a decrease in the mRNA expression of the enzyme HMG‐CoA reductase, which is involved in the manufacture of cholesterol. This decrease suggests that the production of cholesterol, which is often elevated in NAFLD, may be inhibited by resveratrol. The clinical trials on resveratrol's impact on NAFLD patients' liver structure have often been too brief to provide significant findings. Taking 150 mg of resveratrol daily for 3 months, for example, did not improve liver fat content or cardio‐metabolic markers in individuals with insulin‐resistant NAFLD who were overweight or obese (Kantartzis et al. 2018). However, the limited bioavailability and dose‐dependent characteristics of resveratrol were overlooked in this study, suggesting that the dosage may be too low.

On the other hand, a different study found that those with NAFLD had an interesting drop in TNF‐α (Huby and Gautier 2022). In light of this, more sample numbers and an extended period of high‐dose, bioavailable resveratrol therapy are needed to confirm the effectiveness of this drug for liver modification in patients with NAFLD. Additionally, while resveratrol benefits liver health, its potential cytotoxicity in high concentrations should be further investigated to ensure safety and efficacy in therapeutic applications.

## 5 Role of Resveratrol in Hepatocellular Cancer: Focus on Inflammation and Oxidative Stress

Chronic inflammation is a driving force in the development of HCC. Pro‐inflammatory cytokines such as TNF‐α, IL‐6, and IL‐1β activate signaling pathways like NF‐κB and STAT3, which promote tumor cell proliferation, angiogenesis, and metastasis. Resveratrol has been shown to inhibit these inflammatory mediators through several mechanisms. Resveratrol suppresses the activation of NF‐κB, a key transcription factor involved in the expression of genes encoding inflammatory cytokines, chemokines, and adhesion molecules. By blocking NF‐κB signaling, resveratrol reduces the expression of pro‐inflammatory genes that support tumor progression. In both in vitro and in vivo models of HCC, resveratrol decreases the levels of inflammatory cytokines, including IL‐6 and TNF‐α. This helps to reduce the tumor‐promoting microenvironment and attenuates liver inflammation. Importantly, resveratrol also inhibits the expression of enzymes COX‐2 and iNOS, which are frequently upregulated in HCC and are involved in sustaining inflammation. This broader impact of resveratrol on inflammation in HCC is significant as it helps to reduce the likelihood of malignant transformation.

Oxidative stress, caused by the overproduction of ROS, plays a central role in liver carcinogenesis by inducing DNA damage, lipid peroxidation, and mitochondrial dysfunction. Resveratrol exerts potent antioxidant effects that can counteract these damaging processes. Resveratrol neutralizes ROS, reducing oxidative damage to cellular macromolecules such as DNA, proteins, and lipids. The nuclear factor erythroid 2–related factor 2 (Nrf2) is a transcription factor that regulates the expression of antioxidant response element (ARE)‐driven genes. Resveratrol activates the Nrf2 signaling pathway, leading to upregulation of endogenous antioxidant enzymes such as superoxide dismutase (SOD), catalase, and glutathione peroxidase. This contributes to the detoxification of harmful oxidative metabolites. Resveratrol has been shown to preserve mitochondrial membrane potential and prevent mitochondrial‐mediated apoptotic signaling caused by oxidative stress. By stabilizing mitochondria, resveratrol not only supports cellular energy metabolism but also prevents cancer cell survival mechanisms, offering a reassuring and confident approach to HCC treatment. Importantly, inflammation and oxidative stress are closely interrelated in HCC pathogenesis. ROS can activate inflammatory signaling pathways, while inflammation can induce ROS production. Resveratrol's ability to target both pathways makes it uniquely effective in disrupting this vicious cycle. For instance, by reducing oxidative stress, resveratrol indirectly suppresses NF‐κB activation and vice versa. This dual action not only disrupts the vicious cycle of inflammation and oxidative stress but also holds the potential to restore hepatic cellular homeostasis, offering a promising avenue for HCC treatment.

## 6 Apoptosis

**FIGURE 2:**
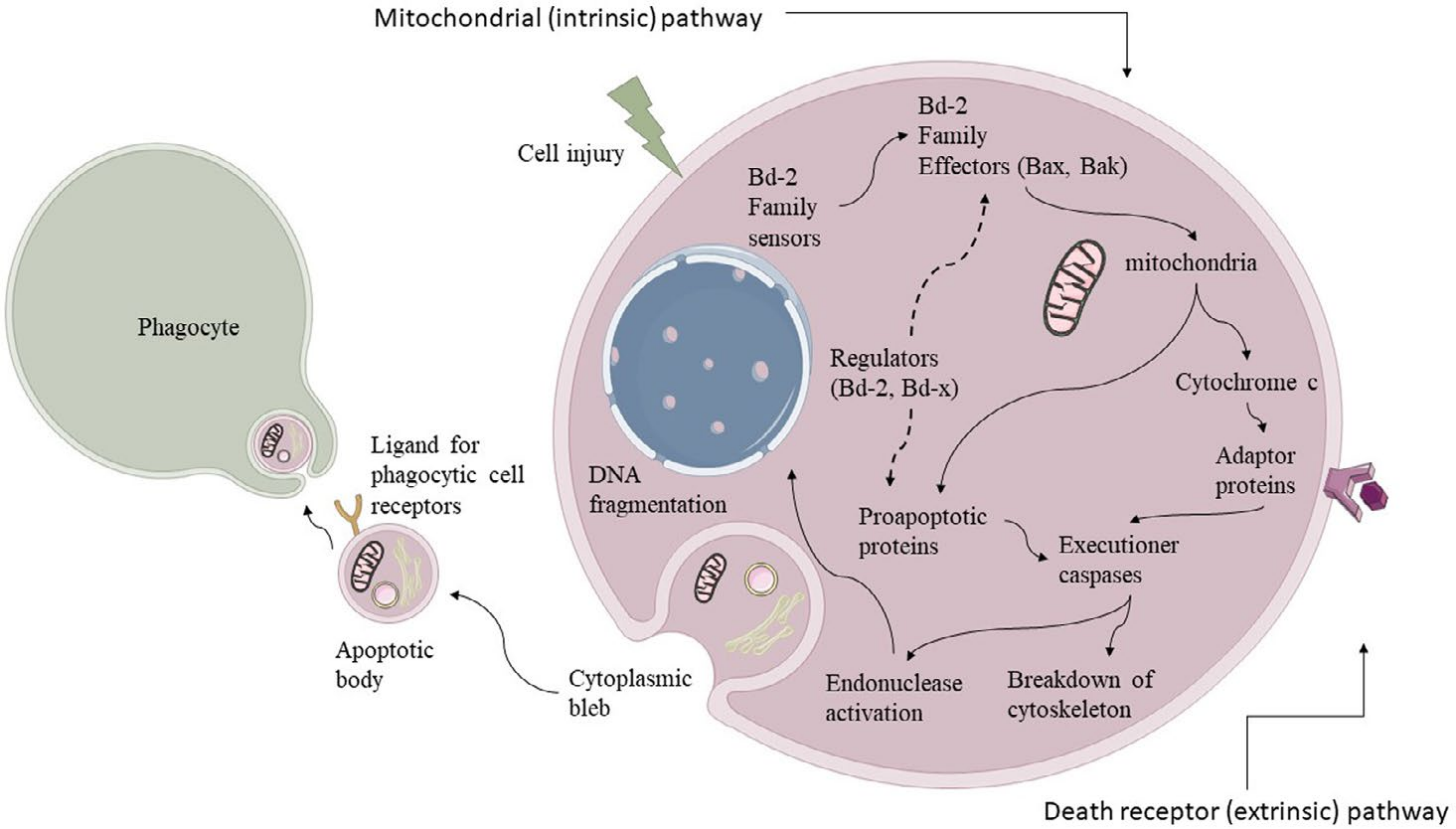
Intrinsic and Extrinsic pathways of apoptosis.

One thoroughly researched and regulated method of cell death is apoptosis. Apoptotic bodies finally form due to certain cell structural modifications, such as cell shrinkage and chromatin condensation, which are characteristics of this highly energetic and closely controlled process (Schwabe and Luedde 2018). Activating intracellular caspases, which are cysteine proteases, typically initiates these biological processes. Upon initial inactivation, caspases require proteolytic cleavage to regain their activity. Endonucleases are then activated, cleaving DNA into short, regular‐sized double‐strand fragments. Both the intrinsic and extrinsic pathways—two processes that initiate apoptosis—are operational in liver cells (Figure 2). The extrinsic pathway starts when surface death receptors are activated by ligands that belong to the tumor necrosis factor (TNF) superfamily (Pistritto et al. 2016). The extrinsic pathway is triggered by the binding of ligands such as TNF‐α, Fas ligand (FASL), and TNF‐related apoptosis‐inducing ligand (TRAIL) to receptors such as TNF receptor 1 (TNFR1), Fas (TNF receptor superfamily member 6), death receptor 4 (DR4)/TRAIL‐R1, and death receptor 5 (DR5)/TRAIL‐R2 (Feldstein et al. 2003; Ribeiro et al. 2004). Complex IIa, called the death‐inducing signaling complex (DISC), is a large complex that arises from activating these receptors' cytosolic domain and drawing in other signaling proteins (Guicciardi and Gores 2009). The death‐inducing signaling complex (DISC) assembly leads to the activation of caspase‐8 and facilitates the production of pro‐caspase‐8 homodimers and their auto‐processing. After activating caspase‐8, executioner caspases −3, −6, and −7 carry out the cellular demolition processes. Significantly, the TNFR1/TNF and Fas levels have been elevated in the livers of individuals with NASH, as indicated by several studies (Feldstein et al. 2003; Ribeiro et al. 2004). This increase is associated with the severity of the disease. Interestingly, when the expression of Fas was suppressed using synthetic small interfering RNAs (siRNA) in an experimental NAFLD model, it reduced steatosis and liver injury (Savari et al. 2019). In hepatocytes, caspase‐8‐dependent apoptotic signaling is frequently enhanced by activation of the intrinsic or mitochondrial route. At this amplification stage, caspase‐8 cleaves the BH3‐interacting domain death agonist (Bid), resulting in tBid, a truncated form. After translocating to the mitochondria, it causes the mitochondrial outer membrane permeabilization (MOMP) process, which releases pro‐apoptotic substances, including cytochrome c, into the cytosol (Hatano et al. 2000; Li et al. 2002). By attaching to apoptotic protease‐activating factor 1 (Apaf‐1), cytochrome c promotes the formation of an apoptosome and the proteolytic activation of caspase‐9 in the cytoplasm. Caspase‐9 then triggers caspase‐3 and the subsequent stages of apoptosis (Hengartner 2000). The intrinsic apoptotic pathway may also be triggered by various intracellular events, including ROS, DNA damage, and ER stress, all of which result in MOMP. In addition to hepatocyte fate, mitochondrial integrity, and other variables, the Bcl‐2 protein family, with its intriguing role in regulating MOMP, captures our attention. The pro‐apoptotic factors Bak and Bax are necessary for MOMP, although apoptosis is inhibited when the regulatory proteins Bcl‐2, Bcl‐xL, and Mcl‐1 bind to Bak and Bax.

Furthermore, BH3‐only proteins like Bid, Bim, Bad, and Noxa, which operate as apoptotic sensors in response to various apoptotic stimuli, can directly or indirectly activate pro‐apoptotic effectors. Strangely, it has been shown that NASH patients' livers overexpress both the Bax and Bcl‐2 proteins (Ramalho et al. 2006). This dysregulation, which disrupts the balance between pro‐apoptotic and anti‐apoptotic signals, can lead to increased cytotoxicity, contributing to liver damage and the progression of liver diseases. This imbalance in NASH should raise concerns about the potential for increased liver damage.

### 6.1 Induction of Apoptosis by Resveratrol

Apoptosis plays a critical part in cancer treatment, and rationally manipulating this process can have a significant effect on cancer therapy (Carneiro and El‐Deiry 2020). A unique strategy for cancer treatment may be possible if some drugs, like resveratrol, can control the apoptotic pathway (Jang, Im, and Kim 2022). By modulating apoptosis, these agents may enhance the programmed cell death of cancer cells, leading to improved therapeutic outcomes (Jang, Im, and Kim 2022). Moreover, resveratrol's potential to induce cytotoxicity in cancer cells further underscores its promise as an effective treatment, inspiring hope for the future of cancer treatment. The modulation of apoptosis triggers cell death and contributes to the cytotoxic effects that can help eliminate malignant cells. Thus, the potential of resveratrol or related chemicals to modulate the apoptotic pathway offers a viable route for creating novel cancer therapeutic approaches. The several apoptotic molecular targets that resveratrol regulates in HCC are compiled in Table 1.

**TABLE 1:** Molecular targets of resveratrol‐induced apoptosis in hepatocellular cancer.

Authors	Model (in vivo, in vitro)	Mechanism	Downregulation	Upregulation	Type of cancer
Yang et al. (2018)	SMMC7721 cells	Apoptosis	STAT1	SIRT1, p‐STAT1	HCC
Parekh et al. (2011)	HepG2 cell line	Apoptosis	Cyclin D1, p38 MAP kinase, Akt/PKB, and Pak1	ERK	HCC
Zhang et al. (2018)	MHCC97‐H cell	Apoptosis	p62, ratio of p‐Akt/Akt	Beclin1, ratio of LC3 II/I, p53	HCC
Dai et al. (2015)	HCC‐LM3, SMMC‐7721, Bel‐7402, QSG‐7701, LO2 cells	Apoptosis	HK2	caspase‐3, PARP1 cleavage, Bax	HCC
Leischner et al. (2021)	HCC; HepG2, Hep3B	Apoptosis	—	—	HCC
Chai et al. (2017)	HepG2, Bel‐7402 and SMMC‐7721	Apoptosis	PCNA, Bcl‐2, Bcl‐2/Bax ratio, PARP, caspase‐3 and ‐7, p‐PI3K, p‐AKT, Ac‐FOXO1, p‐FOXO3a	Bax, PARP1 cleavage, SIRT1, SIRT1 activity, DCL	HCC
Park et al. (2017)	Huh7‐HBx	Apoptosis	Survivin, Akt, cyclin D1	—	HCC
Zhang et al. (2013)	Diethylnitrosamine (DENA)‐induced liver cancer	Apoptosis	MLCK	—	HCC
Karabekir and Özgörgülü (2020)	Diethylnitrosamine (DENA)‐induced liver cancer	Apoptosis	—	Bax/Bcl‐2 and p53	HCC
Liu et al. (2016)	HepG2	Apoptosis	p‐Akt and p‐FoxO3a	FoxO3a, Bim	HCC
Liu, Sun, and Li (2018)	C3A, SMCC7721 and LO2 cells	Apoptosis	ASCT2	cytochrome c, caspase‐9, caspase‐3	HCC
Dai et al. (2020)	Diethylnitrosamine (DENA)‐induced liver cancer	Apoptosis	Bcl‐2	Bax, Bax/Bcl‐2 ratio.	HCC
Dai et al. (2020)	HepG2 and Hep3B	Apoptosis	MARCH1, p‐AKT	PTEN	HCC

#### 6.1.1 Effect of Resveratrol on SIRT

Sirtuin is an enzyme that depends on NAD+ for action. It belongs to the class III histone deacetylases (HDACs). Because these enzymes remove acetyl groups from various substrates, they are essential for many fundamental cellular processes, including metabolism, senescence, aging, stress response, and cancer (Kang et al. 2022). SIRT1‐7 is the name for the seven isoforms of sirtuin found in mammals. These isoforms have a variety of cellular locations and molecular targets in addition to being engaged in a broad range of cellular pathways and functions. More specifically, SIRT1 usually protects cells from oncogenic transformation. However, SIRT1's enzymatic activity can potentially promote cancer progression by inhibiting molecules that lead to apoptosis, contributing to cytotoxicity under certain conditions. Because its actions can either promote or hinder cancer formation depending on the particular cellular milieu, signaling pathways, or disease targets involved, SIRT1 plays a dual function in cancer (Ong and Ramasamy 2018).

The possible effects of resveratrol on SIRTs have been the subject of much research (Ong and Ramasamy 2018). By removing acetyl groups from target proteins through their enzymatic activity, SIRT proteins are known to control gene expression and protein function (Nandave et al. 2023). The most researched member of the SIRT family, SIRT1, has been demonstrated to be activated by resveratrol. Resveratrol‐induced SIRT1 activation has been connected to several beneficial impacts on health and metabolism. Resveratrol is believed to be able to boost SIRT1 expression and activity, which in turn causes target proteins involved in energy metabolism, such as forkhead box O1 (FOXO1) and peroxisome proliferator‐activated receptor gamma coactivator 1‐alpha (PGC‐1α), to be deacetylated and activated. These proteins can be activated to improve insulin sensitivity, mitochondrial biogenesis, and the metabolism of fats and carbohydrates. Resveratrol has been demonstrated to have effects on additional SIRT family members in addition to SIRT1. It can, for instance, activate SIRT3, which is mainly found in mitochondria and has been connected to the control of oxidative stress and mitochondrial activity (Wang et al. 2023). Better mitochondrial activity and defense against age‐related illnesses have been linked to resveratrol's activation of SIRT3.

#### 6.1.2 Effect of Resveratrol on AKT

AKT is a serine/threonine kinase known as protein kinase B (PKB). It is essential for many physiological processes, including glycogen metabolism, growth, proliferation, apoptosis, and cell survival (Revathidevi and Munirajan 2019). It is a critical part of the AKT/phosphoinositide 3‐kinase (PI3K) signaling pathway. Numerous stimuli can activate AKT, such as growth hormones, inflammation, DNA damage, and phosphoinositide‐dependent kinase (PDK) or PI3K activation. Upon activation, AKT phosphorylates and modulates several downstream effectors, including glycogen synthase kinase 3 beta (GSK3β), mammalian target of rapamycin (mTOR), and forkhead box protein O1 (FOXO1). These effectors play a role in controlling cells' metabolism, growth, and survival.

In the context of cancer, AKT is abnormally elevated or activated in several malignancies, including pancreatic, ovarian, and lung cancer. This abnormal AKT signaling is frequently linked to higher cancer cell proliferation, improved survival, and resistance to apoptosis. Consequently, the modulation of AKT activity has implications for enhancing cytotoxicity against cancer cells and improving therapeutic strategies. As a result, focusing on AKT has become a viable therapeutic approach for cancer prevention and treatment (Song et al. 2019).

#### 6.1.3 Effect of Resveratrol on Tumor Suppressor p53

The protein p53 is essential for controlling cellular responses to DNA damage and preventing tumor development (Liu et al. 2019). Specific genes involved in critical physiological functions, including stopping the cell cycle, repairing DNA damage, and starting apoptosis, are produced when p53 is active (Zhao and Sanyal 2022). An important function of p53 is its ability to promote apoptosis by activating genes that induce cell death. These genes, which belong to the Bcl‐2 family and include Noxa, Bax, and PUMA, assist in preserving the equilibrium between elements that encourage and obstruct apoptosis in cells.

Furthermore, p53 promotes the activation of caspases, which are essential enzymes for carrying out apoptosis. It achieves this by upregulating the expression of genes that trigger apoptosis, including Fas, Bid, DR5, and Apaf‐1, while inhibiting genes that stop apoptosis, such as surviving (Ou et al. 2022). After it is created, p53 undergoes several molecular changes that closely regulate its function. The stability, location within the cell, and interactions with other proteins are all impacted by these modifications, which include phosphorylation, acetylation, methylation, neddylation, and ubiquitination. Ultimately, these changes affect p53's capacity to control gene expression and perform its functions (Liu et al. 2019). In the context of cytotoxicity, p53's regulatory role becomes even more crucial, as it helps to determine the fate of cells exposed to harmful agents, including those that induce oxidative stress or DNA damage. p53 is essential for preserving the stability of the genome and halting the onset and spread of cancer because it regulates the expression of genes related to cell cycle regulation, DNA repair, and apoptosis. However, in many cancer types, p53 can become dysregulated or altered, impairing its capacity to prevent tumor development and causing reduced function.

### 6.2 Role of Resveratrol‐Induced Apoptosis in Hepatocellular Cancer

Apoptosis dysregulation is a defining feature of all cancers, including HCC (Kouroumalis et al. 2023). Inducing apoptosis in cancer cells is an important therapeutic strategy for inhibiting tumor growth and improving treatment outcomes. Resveratrol has been found to modulate multiple signaling pathways involved in apoptosis regulation in HCC cells (see Table 1). The PI3K/Akt signaling pathway, which is typically dysregulated in HCC and increases cell survival and resistance to apoptosis, is one of the major routes that resveratrol targets. Resveratrol has been demonstrated to inhibit Akt activity, which suppresses anti‐apoptotic proteins and activates pro‐apoptotic factors (Kouroumalis et al. 2023). Resveratrol can influence the PI3K/Akt pathway and other signaling pathways linked to apoptosis, including the Wnt/β‐catenin system, the MAPK pathway, and the NF‐κB pathway. Resveratrol can decrease the development of HCC cells and encourage apoptosis by modifying these mechanisms. Additionally, resveratrol has been shown to have anti‐inflammatory and antioxidant qualities, which may help explain why it has anticancer benefits. The onset and advancement of HCC are strongly linked to oxidative stress and persistent inflammation. Thanks to its anti‐inflammatory and antioxidant properties, resveratrol can help prevent inflammation, lessen cellular damage, and make it harder for cancer cells to survive.

In the study conducted by Parekh et al. (2011), significant findings were revealed about the signaling pathways connected to cell survival and proliferation and the molecular processes underpinning resveratrol's action and impacts on cyclin D1 expression. The study's examination of the chemopreventive effects of resveratrol on the expression of cyclin D1 in HepG2 cells, a subset of cells associated with liver cancer, provided valuable insights. The results showed that resveratrol therapy decreased the expression of cyclin D1, p38 MAP kinase, Akt/PKB, and Pak1, as well as their amounts, linking the downregulation of these pathways to the growth‐inhibitory effects of resveratrol on HepG2 cells (Parekh et al. 2011).

Furthermore, the study by Leischner et al. (2021) investigated the impacts of the cis and trans forms of the compound resveratrol on the growth and survival of four different types of human cancer cells. The outcomes showed that trans‐resveratrol had more cytotoxicity and antiproliferative effects in all examined tumor cell lines than cis‐resveratrol, suggesting its potential in enhancing cancer cell death. This finding is particularly intriguing and warrants further investigation.

Additionally, the researchers looked at the function of the p53 tumor suppressor protein, which is essential for controlling cell division and averting the development of tumors. The influence of p53 on resveratrol‐induced cell death was assessed using cell lines with varying p53 status, such as wild‐type, p53‐deleted, and p53‐mutated cells. The study found that tumor cells with functioning p53 were more susceptible to resveratrol‐induced cell death. This suggests that resveratrol's effects on cell death are influenced by the presence and functionality of p53, a key regulator of cell division and tumor suppression (Leischner et al. 2021).

The PI3K/AKT signaling system is essential for the growth, survival, neovascularization, and proliferation of cells. AKT, sometimes called protein kinase B, is an important PI3K pathway downstream of target kinase. Numerous anti‐apoptotic actions of activated AKT support the proliferation and survival of cancer cells. One of its leading roles is to prevent the release of cytochrome c and other substances that cause apoptosis from the mitochondria. The release of cytochrome c, an essential mediator of the intrinsic apoptotic pathway, sets off a series of processes that culminate in cell death. Activated AKT prevents apoptosis and increases cell survival by blocking the release of cytochrome c. Resveratrol's ability to influence the PI3K/AKT pathway is significant as it targets a key pathway involved in cell growth and survival, providing a potential mechanism for its anticancer effects. AKT activation can also prevent pro‐apoptotic proteins like caspase‐9 and BAD (Bcl‐2‐associated death promoter) from acting. As a member of the Bcl‐2 protein family, BAD stimulates apoptosis by counteracting Bcl‐2 and Bcl‐XL's anti‐apoptotic actions. When AKT phosphorylates BAD, it is isolated from pro‐apoptotic proteins and cannot function. Cell survival is enhanced, and this phosphorylation process inhibits apoptosis. It has been shown that resveratrol inhibits the PI3K/AKT pathway's ongoing activation, which causes apoptosis to be induced in a variety of cancer cell types. FoxO3a is phosphorylated in response to AKT activation, and FoxO3a functions as a target downstream of AKT. This phosphorylation process causes FoxO3a to translocate from the nucleus to the cytoplasm, where it inactivates. On the other hand, FoxO3a's dephosphorylation is encouraged when AKT is blocked, which makes it easier for FoxO3a to translocate back into the nucleus. In the research of Chai et al. (2017), it was observed that resveratrol treatment led to a notable inhibition of the constitutively elevated levels of phosphorylated PI3K/AKT in HepG2 cells. Furthermore, these cells showed a significant decrease in phosphorylated FoxO3a following resveratrol therapy. AKT uses a variety of targets, including the caspase proteases and the Bcl‐2 protein family, to carry out its anti‐apoptotic actions. The members of the Bcl‐2 family tightly regulate the intrinsic apoptotic pathway. AKT may phosphorylate and activate Bcl‐2 and Bcl‐XL, two pro‐survival Bcl‐2 family members. By blocking the cytochrome c release from mitochondria, these proteins stop caspase proteases from activating and causing apoptosis. Besides its impact on the Bcl‐2 family, AKT can directly impede caspase proteases, essential for the apoptotic process. An initiator caspase implicated in the intrinsic apoptotic process, caspase‐9, can be phosphorylated and inactivated by activated AKT. AKT prevents downstream caspase‐3 and caspase‐7 from activating and carrying out apoptosis by blocking caspase‐9. The Bcl‐2/Bax ratio was shown to fall significantly in response to resveratrol administration in the study by Chai et al. (2017), suggesting a change in favor of increasing apoptosis. Furthermore, resveratrol triggered the activation of PARP, caspase‐3, and caspase‐7, resulting in the cleavage of PARP in HCC cells. These results imply that the mitochondrial route is the mechanism via which resveratrol induces apoptosis in HCC cells (Chai et al. 2017). Cytochrome c is released from the mitochondria by this process, which also involves the activation of caspases and the cleavage of target proteins, including PARP, which eventually results in cell death.

SIRT1 interacts with several proteins, including the NF‐κB family, FoxOs (Forkhead box O transcription factors), and members of the p53 family, to regulate processes related to cell death and survival (Blander and Guarente 2004). About apoptosis, SIRT1 can regulate the function of p53, a well‐known tumor suppressor protein. SIRT1 suppresses p53's pro‐apoptotic activities via deacetylating and inhibiting it. Through p53 deactivation, SIRT1 stimulates cell proliferation and prevents apoptosis (Blander and Guarente 2004). SIRT1 interacts with FoxOs, a family of transcription factors involved in apoptosis and cell cycle control, in addition to p53. FoxOs are deacetylated and activated by SIRT1, facilitating their translocation to the nucleus and activating genes linked to cell cycle arrest and death. This process implies that SIRT1 may activate FoxOs, leading to cell death (Blander and Guarente 2004). However, SIRT1 can potentially influence cell viability by interacting with the transcription factors of the NF‐κB family. The regulation of immunological responses and cell survival is associated with NF‐κB. SIRT1 decreases the transcriptional activity of NF‐κB by deacetylating and inhibiting it. Under some circumstances, SIRT1's inhibition of NF‐κB can reduce genes that support survival and inflammation, which in turn can promote cell death (Blander and Guarente 2004). Research has shown that resveratrol can stop cancer cells from proliferating both in vivo and in vitro. Significantly, the activity of SIRT1, a protein known as sirtuin 1, is required for resveratrol to have an inhibitory impact on cell proliferation (Yang et al. 2013). It was shown in the Chai et al. study that resveratrol treatment of HCC cells led to a significant upregulation of SIRT1 expression (Chai et al. 2017). FoxO1 has been identified as a critical protein that controls the expression of genes linked to apoptosis in cancer cells (Fu and Tindall 2008). As a transcription factor belonging to the Forkhead box O (FoxO) family, FoxO1 is essential for controlling several biological processes, including apoptosis, DNA repair, and cell cycle arrest (Fu and Tindall 2008). Researchers observed that siRNA knockdown enhanced FoxO1 acetylation (Ac‐FoxO1) and prevented ROS‐induced apoptosis in mouse embryonic stem cell research (Chae and Broxmeyer 2011). Research by Chai et al. noted that resveratrol therapy led to a noteworthy reduction in FoxO1 and Ac‐FoxO1 expressions (Chai et al. 2017). Resveratrol also activated SIRT1, which went hand in hand with this action.

Moreover, SIRT1 is crucial in negatively regulating cancer cells' PI3K/AKT pathway. It achieves this by deacetylating and modulating the expression of several key components in this pathway. Specifically, SIRT1's deacetylation of PTEN enhances its phosphatase activity, downregulating the PI3K/AKT pathway (Ikenoue et al. 2008). This process enhances the tumor suppressor activity of PTEN, inhibiting AKT activation (Wang et al. 2015). The study conducted by Chai et al. (2017) found that treating HepG2 cells with resveratrol resulted in an upregulation of SIRT1 levels. Important elements of the PI3K/AKT signaling pathway, PI3K and AKT, showed a corresponding drop in phosphorylation levels following this rise in SIRT1 expression. The results indicated that the suppression of PI3K and AKT phosphorylation within the PI3K/AKT pathway was partly due to the upregulation of SIRT1 produced by resveratrol. The results indicated that the suppression of PI3K and AKT phosphorylation within the PI3K/AKT pathway was partly due to the upregulation of SIRT1 produced by resveratrol.

Hepatitis B virus (HBV) infection that persists over time is the primary cause of HCC. Because HBV‐induced HCC is still highly prevalent, even with HBV vaccines available, effective therapies are still desperately needed. In the course of HCC formation, the HBV X protein (HBx) is essential. Because of its anti‐apoptotic properties, it has been identified as the primary factor in the growth of tumors. The research done by Park et al. (2017) offers proof of resveratrol's capacity to impede HBV‐induced HCC. The results imply that resveratrol can inhibit the Akt signaling pathway, which can reduce cell viability, cause G1 cell cycle arrest, and alter the expression of cyclin D1. The BIRC5 gene (Altieri 2006) encodes survivin, an inhibitor of apoptosis proteins (Eckelman et al. 2006). It regulates cell division, modifies cell death, and is a stress response factor contributing to cell survival and proliferation. It performs various functions in cellular processes (Altieri 2006; Eckelman et al. 2006). Through controlling gene expression and protein interactions, survivin inhibits apoptosis through several mechanisms (Altieri 2008). Its function as an anti‐apoptotic protein involves multiple mechanisms that allow it to modulate cellular processes responsible for cell survival. HBx has been shown to interact with survivin and regulate its expression, contributing to the anti‐apoptotic and pro‐survival effects observed in HCC cells (Zhang et al. 2009, 2005, 2014). On the other hand, resveratrol has been investigated for its ability to downregulate survivin expression in HCC cells, potentially promoting apoptosis and inhibiting tumor growth (Park et al. 2017).

Protein myosin, which is involved in muscle contraction, is phosphorylated by MLCK. Phosphorylation of myosin enables it to interact with actin filaments, leading to muscle contraction. The activity of MLCK is tightly regulated and can be influenced by various signaling pathways and molecules. For example, MLCK can be activated by calcium‐calmodulin complexes, which are formed in response to an increase in intracellular calcium levels. Additionally, protein kinases like Rho‐associated protein kinase (ROCK) and protein kinase C (PKC) can control MLCK by phosphorylating and changing its activity. MLCK may contribute to the onset and spread of cancer (Minamiya et al. 2005). Zhang et al. (2013) found higher MLCK expression in HCC rats' livers than in normal and resveratrol‐treated rats. Elevated MLCK expression was associated with cell proliferation and anti‐apoptotic effects, indicating a potential role for MLCK in promoting tumor growth and survival in the context of HCC (Zhang et al. 2013). One possible mechanism by which resveratrol may downregulate MLCK expression is by inhibiting specific signaling pathways involved in MLCK regulation. Various signaling molecules, including growth factors, cytokines, and intracellular signaling cascades such as MAPK and NF‐κB, can regulate MLCK expression. Resveratrol has been shown to inhibit these signaling pathways in different cellular contexts, and it may interfere with the activation of these pathways, thereby reducing MLCK expression. Resveratrol has also reportedly been shown to possess anti‐inflammatory and antioxidant qualities. The overexpression of MLCK can be attributed to both inflammation and oxidative damage. Resveratrol has the potential to indirectly downregulate MLCK expression by lowering oxidative stress and inflammation.

The E3 ubiquitin ligase MARCH 1 (Membrane‐Associated RING‐CH‐Type Finger 1) possesses a membrane‐associated RING‐CH domain. It contributes to the control of innate immunity. Studies have revealed that HCC tissues and cells express MARCH 1 at much greater levels. It has been discovered that MARCH 1 knockdown causes apoptosis and prevents HCC cells from proliferating, migrating, and invading. As a tumor promoter that controls the β‐catenin/phosphoinositide‐3‐kinase/protein kinase B (PI3K/AKT) signaling pathway, MARCH 1 has been discovered. As such, it may be a promising molecular therapeutic target for HCC. In a study conducted by Parekh et al. (2011), it was observed that resveratrol exhibited a dose‐dependent effect on the expression of MARCH 1 and phospho‐protein kinase B (p‐AKT), leading to their downregulation. This finding underscores the potential of resveratrol as a promising therapeutic target for HCC, offering hope for future treatments.

In contrast, resveratrol increased the expression of phosphatase and tensin homolog deleted on chromosome 10 (PTEN) in both in vitro and in vivo tests. The investigation also showed that PTEN expression rose in response to the MARCH 1 knockdown, suggesting that MARCH 1 and PTEN are regulated similarly. Furthermore, when resveratrol was paired with AKT and PTEN inhibitors, the researchers observed a more marked reduction in MARCH 1 expression compared to the group that received resveratrol alone (Dai et al. 2020).

Resveratrol may make HCC cells more susceptible to the effects of chemotherapy, increasing the medications' capacity to eradicate cancer cells (Liu, Peng, et al. 2018). This synergistic effect can improve treatment outcomes by allowing lower doses of chemotherapy drugs to be used, reducing their toxicity. The exact mechanisms by which resveratrol enhances the cytotoxic effects of chemotherapy drugs in HCC are still under investigation. One significant challenge in HCC treatment is the development of drug resistance, which limits the effectiveness of chemotherapy. It has been discovered that resveratrol reverses or suppresses drug resistance pathways, hence sensitizing HCC cells to chemotherapeutic medicines. It can inhibit drug efflux pumps, like P‐glycoprotein, which remove drugs from cancer cells and reduce their concentration inside the cells. By inhibiting these pumps, resveratrol increases the accumulation of chemotherapy drugs within the cells, making them more susceptible to the drugs' cytotoxic effects. This potential of resveratrol to enhance chemotherapy efficacy is a source of encouragement and inspiration in the fight against HCC.

Indeed, resveratrol has been found to impact various signaling pathways involved in apoptosis, which is a critical mechanism of action for many chemotherapy drugs. Bcl‐2 and Bax proteins, crucial for controlling apoptosis, can express abnormally and be affected by resveratrol. Cytochrome c can also be released from mitochondria into the cytosol (Liu, Peng, et al. 2018; Ma et al. 2015). It enhances the activation of pro‐apoptotic factors and inhibits anti‐apoptotic proteins, increasing the likelihood of apoptosis in HCC cells. This synergistic effect between resveratrol and chemotherapy drugs promotes cell death and enhances cytotoxicity. Resveratrol also affects cell cycle regulators, such as cyclins and cyclin‐dependent kinases (CDKs), which can cause cell cycle arrest, especially during the G1 phase. This cell cycle arrest enhances the cytotoxic effects of chemotherapy drugs that target dividing cells. When resveratrol is combined with cisplatin, it has been observed to enhance the production of ROS within cancer cells. While cisplatin (CDDP) is widely acknowledged as a highly effective chemotherapy drug for HCC, the emergence of resistance to cisplatin can significantly impede its efficacy in treatment (Shaaban et al. 2014). It has been proposed that the enhancement of cisplatin toxicity by resveratrol may be associated with increased ROS‐induced DNA damage in HCC (Liu, Peng, et al. 2018). The co‐administration of cisplatin and resveratrol can lead to elevated ROS levels, which can cause various types of DNA damage, such as oxidative DNA alterations, double‐strand breaks, and single‐strand breaks. The accumulation of DNA damage caused by increased ROS production can overwhelm the DNA repair mechanisms of cancer cells, leading to persistent DNA lesions.

Persistent damage to DNA can trigger response pathways to DNA damage, which includes phosphorylating H2AX (γH2AX). One of the most reliable indicators of DNA damage and double‐strand breaks is the development of γH2AX foci (Liu, Peng, et al. 2018). The DNA damage resulting from the generation of ROS by resveratrol triggers the intrinsic apoptotic pathway. Cytochrome c is released, caspase‐9 is activated, and caspase‐3 is activated as a result. These sequential events ultimately induce cell death and amplify the toxic effects of cisplatin (Liu, Peng, et al. 2018). The amino acid transporter ASCT2 (Alanine‐Serine‐Cysteine Transporter 2) is essential to cellular metabolism. It is involved in the absorption of glutamine, a nutrient essential for developing and proliferating cancer cells. ASCT2 is often upregulated in various cancers, including HCC. Increased invasiveness, tumor growth, and treatment resistance have all been linked to its overexpression. Liu, Peng, et al. (2018) constructed a recombinant expression plasmid to facilitate high expression of ASCT2 in C3A and SMCC7721 cells, which are HCC cell lines. By introducing this plasmid into the cells, they artificially increased the levels of ASCT2 expression. Liu, Peng, et al. (2018) found that resveratrol could significantly enhance the anticancer effects of the chemotherapy drug cisplatin against HCC cells. However, this enhancing effect of resveratrol was drastically reduced when the HCC cells expressed high levels of the ASCT2 protein. It was initially believed that by downregulating ASCT2 expression, resveratrol and cisplatin would increase their antitumor actions. However, the study showed this increase inhibited when ASCT2 was significantly expressed. This highlights the significance of ASCT2 as a potential therapeutic target for HCC management. In Yang et al. (2018), the researchers sought to determine if the naturally occurring substance resveratrol may improve the efficiency of interferon‐a (IFN‐a) in preventing the development and triggering death of HCC cells. The investigation results demonstrate that SMMC7721 cells, a subset of HCC cells, experience growth inhibition and apoptosis due to IFN‐a therapy. Supplementing with resveratrol greatly amplifies these effects, whereas using EX527 to disrupt SIRT1, a protein implicated in the SIRT/STAT1 pathway, decreases them. In addition to phosphorylating STAT1, resveratrol also activates SIRT1. Subsequent research indicates that STAT1 overexpression enhances the combined anticancer effects of resveratrol and IFN‐a, while STAT1 deficiency decreases these effects. According to the study, resveratrol may activate the SIRT/STAT1 pathway, improving HCC's response to IFN‐a therapy (Yang et al. 2018).

Because it catalyzes the critical step in glycolysis that turns glucose into glucose‐6‐phosphate, HK2 is an essential component of the glycolytic pathway. Even with enough oxygen, HK2 is increased in cancer cells, particularly HCC, and encourages the transition toward aerobic glycolysis. Because of this change in metabolism, cancer cells can grow more quickly and create more energy. Anticancer treatments targeting HK2 are thought to have the ability to interfere with aerobic glycolysis and stop the spread of the illness. In their study, Dai et al. (2015) demonstrated that resveratrol sensitizes HCC cells that exhibit aerobic glycolysis to undergo apoptosis. This indicates that resveratrol has a pro‐apoptotic effect on HCC cells relying on aerobic glycolysis for energy production.

Furthermore, the researchers discovered that resveratrol amplifies the inhibitory impact of sorafenib, a medication frequently used to treat HCC. In HCC cells, the combination of resveratrol and sorafenib showed enhanced efficacy in preventing tumor development and encouraging apoptosis. Hexokinase 2 (HK2) expression in HCC cells was found to be downregulated in response to the combined treatment of resveratrol and sorafenib (Dai et al. 2015).

### 6.3 Induction of Apoptosis by Resveratrol in NAFLD

A growing body of preclinical evidence supports the anti‐apoptotic potential of resveratrol in the management of NAFLD (see Table 2), mainly through modulation of oxidative stress, inflammatory signaling, and mitochondrial‐mediated apoptotic pathways. Yuan et al. (2022) provided mechanistic insight showing that RES attenuates lipotoxicity in oleic acid‐induced L02 hepatocytes and HFD‐fed mice by upregulating Bmi‐1, a protein known to regulate oxidative stress and mitochondrial integrity. This upregulation led to a marked decrease in cleaved caspase‐3 and p53 levels—both pro‐apoptotic markers—alongside an increase in the anti‐apoptotic protein Bcl‐2. When Bmi‐1 was silenced using siRNA, resveratrol lost its protective effect, confirming Bmi‐1 as a central mediator of RES's cytoprotective action.

**TABLE 2:** Resveratrol‐mediated modulation of apoptosis in NAFLD.

Author (Year)	Dosage of resveratrol	Duration	Experimental model	Mechanistic role	Apoptosis marker changes	Key findings
Yuan et al. (2022)	Not specified	OA‐induced (in vitro); HFD (in vivo)	L02 cells (in vitro, OA‐induced) and C57BL/6J mice (in vivo, HFD)	↑ Bmi‐1 expression → ↓ ROS, ↑ SOD, ↓ cleaved caspase‐3, ↓ p53, ↑ Bcl‐2	↓ cleaved caspase‐3, ↓ p53, ↑ Bcl‐2	Resveratrol reduces lipotoxicity by upregulating Bmi‐1. Protective effects abolished by Bmi‐1 siRNA, confirming Bmi‐1 as key in RES‐mediated cytoprotection.
Hajighasem et al. (2018a)	Not specified (administered orally)	Duration not clearly stated	Elderly rats with NAFLD + RSV, exercise, or both	↑ Sirt1, ↑ LXR, ↑ FXR expression; ↓ AST, ALT, ALP; ↑ lipid profile (↓ TG, LDL, ↑ HDL)	↓ TUNEL‐positive apoptotic cells	Resveratrol improved gene expression and liver function; combination with interval/continuous exercise showed stronger anti‐apoptotic and metabolic effects.
Hajighasem et al. (2019)	Not specified	Duration not specified (chronic model)	Old rats with NAFLD; 7 groups: control, RSV, continuous/interval exercise, combos	↓ MDA, ↓ TNF‐α; ↑ catalase, ↑ SOD, ↑ IL‐10; reduced oxidative stress and inflammation	↓ Apoptosis: RSV (17.12%), RSV + interval (10.74%), RSV + continuous (14.85%)	Resveratrol alone has antioxidant, anti‐apoptotic, and anti‐inflammatory effects; combination with exercise improves outcomes more significantly in NAFLD.
Hajighasem et al. (2018b)	Not specified	Duration not specified (training protocol)	Rats with NAFLD induced (method not stated)	Resveratrol ± continuous/interval exercise ↓ lipid profile abnormalities, modulates apoptosis	↓ Bax, ↑ Bcl‐2 (*p* < 0.001)	Resveratrol alone improves lipid profile and apoptosis; combination with continuous/interval training enhances protective effects more significantly than RSV alone
Ghasem et al. (2019)	Not specified	Not stated	NAFLD in old rats via dietary induction	↓ Lipids (↓ LDL, TG, Chol; ↑ HDL); ↑ Bcl2, ↓ Bax	↓ Bax, ↑ Bcl2	Resveratrol and exercise reduced apoptosis and improved lipid profiles; combined therapy (RSV + exercise) was more effective than RSV alone.
Tiao et al. (2018)	50 mg/kg/day in water	From post‐weaning to PND 120	Offspring of rats fed maternal + postnatal high‐fat diets	Modulates renin‐angiotensin system (RAS), ↑ SIRT1, leptin, ↓ oxidative stress, apoptosis, regulates lipid metabolism	↓ Apoptosis (exact markers not specified)	Resveratrol prevents NAFLD in offspring by reducing hepatic steatosis, improving metabolic signaling, and modulating RAS components including ↑ ACE2, ↓ ACE1/AT1R.
Zhang et al. (2025)	Not specified	Not stated	HFD + DEN and AKT/Ras‐induced HCC in mice with steatosis	↓ ROS, ↓ DNA damage, ↓ GST‐pi → ↓ p‐JNK/p‐p38 → ↑ HCC progression	↓ p‐JNK, ↓ p‐p38; apoptosis reduced in HCC cells	Resveratrol reduces steatosis but promotes HCC progression via inhibition of the GST‐pi‐MAPK axis; highlights caution in antioxidant use in fatty liver populations.
Li et al. (2025)	Not specified (likely oral or IP)	6 weeks HFD + intervention phase	NAFLD‐induced Wistar rats (high‐fat diet)	↓ Activin A and TGF‐β (fibrosis/apoptosis‐related cytokines); Resveratrol + aerobic exercise modulated cytokine and metabolic profiles	↓ Activin A & TGF‐β (pro‐apoptotic/fibrogenic cytokines)	Resveratrol, especially with aerobic exercise, reduced inflammation, apoptosis markers (Activin A, TGF‐β), and improved hepatic function in NAFLD rats.

Similarly, several studies by Hajighasem, Farzanegi, and Mazaheri (2018); Hajighasem et al. (2018a, 2018b, 2019) explored the combinatorial effect of RES and exercise in NAFLD models in aged rats. These studies showed that RES alone significantly reduced apoptosis by altering the expression of mitochondrial apoptotic regulators—downregulating Bax and upregulating Bcl‐2 (*p* < 0.001). Notably, combining RES with either continuous or interval exercise training further enhanced these protective effects, reducing apoptosis even more effectively (as quantified by TUNEL assay percentages: RES alone 17.12%, RES + interval training 10.74%, RES + continuous training 14.85%). This suggests that lifestyle interventions may potentiate the anti‐apoptotic benefits of RES.

Ghasem et al. (2019) extended these findings by demonstrating that RES combined with exercise significantly improved lipid profiles (↓ LDL, TG, Cholesterol; ↑ HDL) while modulating apoptotic gene expression (↓ Bax, ↑ Bcl‐2). These effects highlight RES's role in restoring metabolic and apoptotic balance in aging NAFLD models. Tiao et al. (2018) offered a developmental perspective, administering RES at 50 mg/kg/day from post‐weaning to postnatal day 120 in offspring of rats fed HFD before and after birth. Their findings revealed that RES not only improved lipid metabolism but also modulated the renin‐angiotensin system (↑ ACE2, ↓ ACE1/AT1R), reduced oxidative stress, and suppressed hepatic apoptosis. These effects were mediated through upregulation of SIRT1 and leptin signaling, further supporting RES's ability to alter systemic and hepatic stress responses from an early developmental stage.

However, caution is necessary in interpreting these effects universally. Zhang et al. (2025) presented a contrasting viewpoint in a murine model combining HFD and diethylnitrosamine (DEN)‐induced HCC. Although RES reduced hepatic steatosis and oxidative DNA damage, it paradoxically promoted tumor progression by inhibiting the GST‐pi‐MAPK axis—suppressing phosphorylation of JNK and p38 MAPK, key mediators of stress‐induced apoptosis. This unintended effect resulted in reduced apoptosis within HCC cells, suggesting that antioxidant therapies like resveratrol could impair the liver's intrinsic tumor surveillance mechanisms under certain pathological conditions. In this regard, Li et al. (2025) added further evidence by showing that resveratrol, particularly in combination with aerobic exercise, significantly decreased hepatic levels of Activin A and TGF‐β, two cytokines implicated in both fibrosis and apoptosis, thus contributing to improved liver histology and function in NAFLD rats. Collectively, these findings emphasize RES's potential as a therapeutic agent in liver disease through apoptosis regulation, though its effects appear highly context‐dependent—protective in early‐stage metabolic liver disease, but potentially harmful in cancer‐prone hepatic environments due to its anti‐apoptotic actions.

## 7 Autophagy

Proteins, organelles, and macromolecules are cytoplasmic components sequestered into autophagosomes during the catabolic process of autophagy. These autophagosomes combine with lysosomes to generate autolysosomes, which are then broken down by lysosomal enzymes (Jang, Im, Choi, and Kim 2022). For synthesis or energy production, the breakdown products are recycled. Often described as a type II programmed cell death process, autophagy can be a natural part of development or response to pathological or physiological stressors. Although controlled, autophagic cell death is essential for preserving cellular homeostasis and encouraging cell survival in the face of stress and food shortage. The ubiquitin‐proteasome system (UPS) and autophagy are two important biological mechanisms that help preserve protein homeostasis in cells and break down proteins. Ubiquitin‐tagged short‐lived proteins are the primary target of the UPS, which is designed to break them down quickly. The process involves the attachment of ubiquitin molecules to target proteins, which then directs them to the proteasome, a large protein complex that degrades the ubiquitinated proteins into smaller peptides. The UPS is highly efficient in degrading individual proteins, particularly those with a short half‐life or misfolded.

Nevertheless, longer‐lived proteins and bigger cellular structures, such as organelles, are broken down by autophagy. Cytoplasmic components are sequestered into double‐membraned vesicles known as autophagosomes as part of the autophagic process. After fusing with lysosomes to generate autolysosomes, these autophagosomes carry the cargo that lysosomal enzymes break down. Specific components, such as broken organelles or protein aggregates, can be specifically targeted by autophagy for breakdown using particular autophagy processes like mitophagy or aggrephagy (Cohen‐Kaplan et al. 2016). In normal cellular conditions, autophagy helps maintain the metabolic equilibrium of cells, ensuring their survival. Once the byproducts have been broken down inside lysosomes, they are discharged into the cytoplasm, where they are recycled to produce energy (Kaur and Debnath 2015). Nevertheless, during challenging circumstances like oxygen deprivation, lack of nutrients, exposure to genotoxic stress, or infection by pathogens, autophagy plays a crucial role in preserving cellular balance. It facilitates the removal of damaged organelles (including mitochondria and endoplasmic reticula) and intracellular infections. It also aids in the elimination of misfolded or aggregated proteins. This method is greatly controlled by getting rid of these components to provide efficient quality control (Gentile et al. 2022). In this context, resveratrol's cytotoxicity may enhance autophagy's overall effectiveness, contributing to its potential therapeutic effects in hepatocellular cancer and NAFLDs.

### 7.1 Types of Autophagy

**FIGURE 3:**
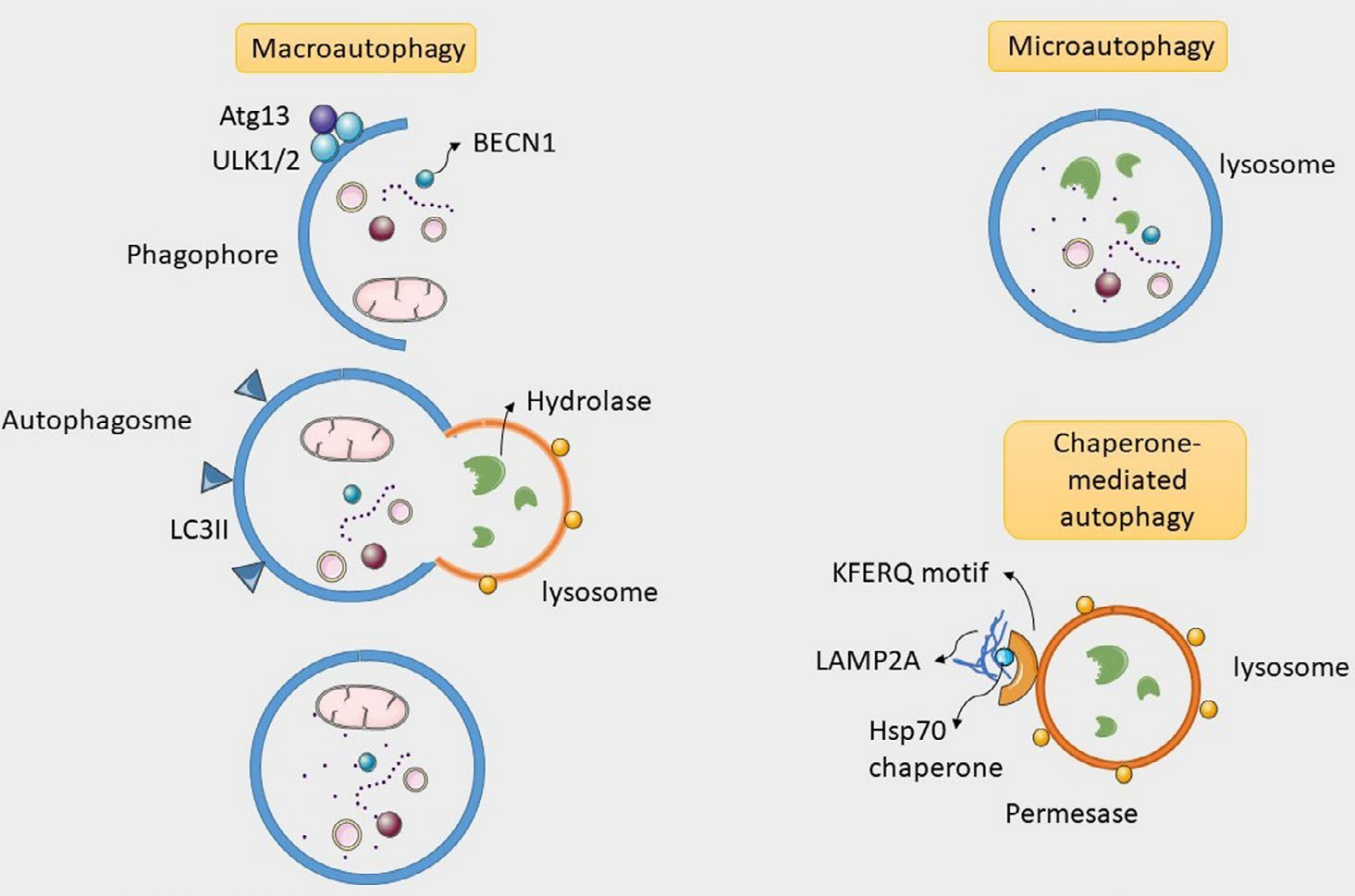
Classification of autophagy. Autophagy can be divided into macroautophagy, microautophagy, and chaperone‐mediated autophagy. BECN1, beclin 1; ULK1/2, UNC‐51‐like kinase 1, UNC‐51‐like kinase 2; LC3 II, microtubule‐associated protein light chain 3 II; Atg 13, autophagy‐related gene 13; LAMP2A, lysosomal‐associated membrane protein 2; Hsp70, heat shock protein 70.

Autophagy may be divided into three categories based on how cellular constituents are transported to the lysosome for breakdown. These include chaperone‐mediated autophagy, macroautophagy, and microautophagy (Figure 3) (Ravanan et al. 2017). The kind of autophagy that has been investigated the most is macroautophagy. Autophagosomes are double‐membrane structures used to sequester various cytoplasmic components, such as protein aggregates and damaged organelles. Afterward, these autophagosomes combine with lysosomes to produce autolysosomes, where lysosomal enzymes break down the contents that have been trapped (Zhao et al. 2021). Microautophagy involves directly engulfing cytoplasmic components by invading or protruding the lysosomal membrane. It occurs when lysosomes directly engulf small portions of cytoplasmic material, leading to their degradation (Zhang et al. 2022). One kind of selective autophagy that targets soluble cytosolic proteins for destruction is chaperone‐mediated autophagy. In CMA, chaperone proteins identify cytosolic proteins with a particular targeting motif and move them to the lysosome, where they translocate across the lysosomal membrane and undergo degradation (Jang, Im, Choi, and Kim 2022).

### 7.2 NAFLD and Autophagy

In the first phases of NAFLD, it is widely acknowledged that autophagy is elevated as a defensive mechanism to avert lipid buildup and preserve cellular equilibrium (Cai et al. 2014). Experimental studies have shown that autophagy activation can promote the breakdown of lipid droplets and enhance lipid clearance in hepatocytes. By removing excess lipids, autophagy helps mitigate lipid‐induced cellular stress, reduce inflammation, and maintain liver cell function. Several factors contribute to the upregulation of autophagy in early‐stage NAFLD. Nutrient excess, such as the increased availability of free fatty acids, can activate autophagy to maintain lipid homeostasis. Insulin resistance, a hallmark of NAFLD, has also been shown to induce autophagy as a compensatory response to metabolic stress. The connection between autophagy and lipid metabolism deepens, and autophagy's function may shift as NASH, a more severe form of NAFLD, advances (Cai et al. 2014). Autophagy dysregulation can occur in later stages of NAFLD, leading to impaired autophagic flux and compromised clearance of damaged organelles and lipids. This dysregulation may contribute to the progression of liver injury and the development of inflammation and fibrosis in NASH. According to Singh et al.'s research, mice fed either a high‐fat or a methionine choline‐deficient (MCD) diet showed decreased liver autophagy. These results suggest lipid accumulation in the liver and the subsequent development of hepatic steatosis may be related to compromised autophagy (Singh et al. 2009). Lipid droplet production in liver cells was considerably enhanced in mice when 3‐maleimidopropionic acid or siRNA was used to silence the ATG5 gene. This implies that increased lipid accumulation in the liver was caused by these treatments that inhibited autophagy (Wang et al. 2010). Conversely, it was discovered that rapamycin, a mTOR inhibitor, increased autophagy. Interestingly, rapamycin also reduced lipid accumulation in both in vitro and in vivo settings (Wang et al. 2010; González‐Rodríguez et al. 2014). These results show that rapamycin treatment‐induced autophagy activation helped decrease hepatic lipid buildup. Moreover, studies have shown that autophagy has a role in controlling the inflammatory response. For instance, in mouse macrophages, disruption of the Atg5 gene led to the suppression of autophagy, which produced increased levels of the pro‐inflammatory cytokine IL‐1β (Ilyas et al. 2016). Furthermore, several studies employing animal models of NAFLD and clinical trials involving NAFLD patients have repeatedly shown that these circumstances decrease autophagy flux, the entire autophagy process (González‐Rodríguez et al. 2014; Yang et al. 2010). These investigations have demonstrated that improving autophagy balance can benefit and lessen the histologic signs and symptoms of fatty liver disease.

There are several ways in which autophagy inhibition in NAFLD might happen (Lavallard and Gual 2014). The mTOR complex controls when autophagy begins and can cause short‐term suppression of autophagy (Lavallard and Gual 2014). When abundant amounts of nutrients are present, such as when there is a high concentration of glucose and amino acids, mTOR functions as a sensor of both nutrients and energy; the production of autophagosomes is hindered by active mTOR, which phosphorylates and inhibits the autophagy‐initiating complex ULK1/2. In NAFLD, autophagy impairment can be lessened by autophagy induction promoted by mTOR inhibition, such as that achieved with rapamycin or other mTOR inhibitors. Conversely, transcription factors, including TFEB and FoxO, may control the long‐term suppression of autophagy in NAFLD (Lavallard and Gual 2014). Genes related to lysosome biogenesis, autophagosome formation, and lysosomal enzyme activity are expressed more often when TFEB is present. Long‐term autophagy suppression in NAFLD can be regulated by TFEB activation. In nutrient‐rich environments, the cytoplasm is where TFEB is mainly found. On the other hand, TFEB translocates to the nucleus in response to food shortage or inhibition of mTOR signaling, which increases the expression of lysosomal and autophagic genes. On the other hand, dysregulated mTOR signaling in NAFLD can prevent TFEB from moving to the nucleus, which lowers autophagy and compromises lysosomal function (Lavallard and Gual 2014).

### 7.3 Role of Resveratrol‐Induced Autophagy in NAFLD

Several studies have explored the role of autophagy in resveratrol's potential to combat NAFLD. Resveratrol, a natural polyphenol found in berries and grapes, has shown promising health benefits, particularly in metabolic diseases like NAFLD. Table 3 outlines the various autophagy‐regulating molecules influenced by resveratrol. The therapeutic effects of resveratrol are closely tied to its modulation of autophagy in NAFLD. For instance, a study by Zhang and colleagues (Zhang et al. 2015) demonstrated that resveratrol administration can enhance autophagy in male C57BL/6 mice fed an MCD diet and in AML12 cells cultured in an MCD medium. This is achieved by upregulating autophagic markers like LC3II and downregulating autophagic negative regulators like p62, suggesting that resveratrol aids in the autophagic process.

**TABLE 3:** Molecular targets of resveratrol‐induced autophagy in NAFLD.

Authors	Model (in vitro, in vivo)	Dosage	Exposure	Mechanism	Up/down expression
Ji et al. (2015)	Mice	100 mg/kg/day or 250 mg/kg/day	28 days	Autophagy	Resveratrol treatment increased LC3‐II levels but decreased P62 expressions.
Ji et al. (2015)	C57BL/6 mice + AML12 hepatocytes under MCD conditions	Mice: 100 or 250 mg/kg/day Cells: 25, 50, 100 μmol/L	Not explicitly stated	Autophagy	Resveratrol ameliorates steatosis and inflammation in MCD‐induced NASH via autophagy modulation. Autophagy is essential for its protective effects.
Li et al. (2014)	HFD‐ mice	50 mg/kg resveratrol	4 weeks	Autophagy	Resveratrol restored the activity of NF‐κB by regulating its inhibitor IκBα
Liu et al. (2020)	NASH mice	30 mg/kg/day	6 weeks	Autophagy	Resveratrol was associated with activation of autophagy, suppression of apoptotic activity, upregulation of lipolytic genes, and reduction of fatty infiltration in limb muscles of NASH mice.
Liu et al. (2020)	Mouse C2C12 cells	40 μM	2 h	Autophagy	Resveratrol alleviated autophagy dysfunction, apoptotic signals, and reduced fusion index and myotube formation.
Ding et al. (2017)	HFD‐Rat	/kg.bw	18 weeks	Autophagy	Resveratrol increased the expression of SIRT1 and markers of autophagy while reducing markers of ER stress in the liver.
Zhang et al. (2015)	HFD‐ mice	Diet containing Resveratrol (0.4%)	weeks	Autophagy	Resveratrol induced autophagy in hepatocytes through the cAMP‐PRKA‐AMPK‐SIRT1 signaling pathway
Zhang et al. (2015)	HepG2 cells	Resveratrol at various concentrations (10, 20, 40, 80 μmol/L)	24 h	Autophagy	Resveratrol induced autophagy in hepatocytes through the cAMP‐PRKA‐AMPK‐SIRT1 signaling pathway
Wang et al. (2022)	HFD‐fed mice, Mouse Leydig cell line TM3 cell	400 mg/kg/day	weeks	Autophagy	Resveratrol reversed LC3II/I, Beclin1, and Atg7

Furthermore, the investigation demonstrated a correlation between resveratrol administrations and reduced inflammatory response and steatosis, or fat buildup in the liver. TNF‐α, IL‐1β, and IL‐6, three pro‐inflammatory cytokines, were downregulated, demonstrating this. It may be inferred from these results that resveratrol has anti‐inflammatory and anti‐steatotic qualities that may be advantageous for handling NAFLD. Moreover, the investigation showed that autophagy was inhibited in AML12 cells treated with the autophagy inhibitor chloroquine. This led to the buildup of oxidative stress in the cells, which ROS represents, and inflammatory response markers (IL‐6, IL‐1β, and TNF‐α). However, after receiving resveratrol therapy, autophagy was restored, which reduced oxidative stress and the inflammatory response. According to these results, resveratrol may bypass chloroquine's suppression of autophagy and reinstate autophagy's beneficial effects on oxidative stress and inflammation (Zhang et al. 2015). This result was ascribed to the cAMP‐PRKA‐AMPK‐SIRT1 pathway being activated. The cAMP‐PRKA‐AMPK‐SIRT1 pathway is a complex signaling cascade that plays a crucial role in cellular energy balance and metabolic regulation. Its activation by resveratrol triggers the autophagy apparatus and improves liver function by encouraging the elimination of lipid droplets (Zhang et al. 2015).

Ding et al. (2017) studied rats with fatty liver disease caused by an HFD to see how resveratrol supplementation and calorie restriction affect hepatic lipid metabolism and the related pathways. Male Wistar rats were used in the research, and they were split into four groups: one for the usual chow diet, one for the HFD, one for the HFD plus resveratrol supplementation, and one for the HFD plus calorie restriction. For 18 weeks, the rats were fed their prescribed diets, and during that time, many metabolic indices, liver histology, and gene and protein expression were evaluated. As demonstrated by the findings, hepatic steatosis and hepatocyte ballooning were only partially avoided by resveratrol administration and calorie restriction. These treatments reduced indicators of ER stress—a physiological reaction to misfolded proteins—in the liver while increasing the expression of SIRT1 and autophagy markers. In addition, the abnormality of lipid metabolism was improved. When comparing the two therapies, calorie restriction outperformed resveratrol in protecting against fatty liver caused by an HFD. Reduced overall calorie intake and body weight were linked to this impact. Based on the study's findings, moderate calorie restriction and resveratrol supplementation can prevent HFD‐induced hepatic steatosis by reducing ER stress and activating the SIRT1‐autophagy pathway (Ding et al. 2017). Li et al. (2014) used a mouse model of NAFLD to investigate resveratrol's therapeutic benefits and underlying mechanisms. The study's conclusions showed that resveratrol therapy improved the assessed NAFLD characteristics. It lessened inflammation and oxidative stress, insulin resistance, glucose tolerance, and enhanced histological features of the liver. The scientists noticed that the administration of resveratrol resulted in the restoration of IκBα, a transcription factor that inhibits NF‐κB, which in turn reduced the activity of NF‐κB. The pathophysiology of NAFLD is linked to NF‐κB, which is known to promote inflammation. According to the study, autophagy and the NF‐κB signaling pathway are two ways resveratrol affects NAFLD and has therapeutic benefits.

In Wang et al. (2022), the researchers investigated the interplay between resveratrol's antioxidant properties, autophagy modulation, and the defense against steroidogenesis decline in the context of oxidative damage and an HFD. They used TM3 cells treated with H_2_O_2_ and resveratrol, as well as male C57BL/6J mice fed a diet enriched with resveratrol. The results showed that resveratrol supplementation reduced steroidogenesis and the mitochondrial dysfunction induced by the HFD. It increased the levels of proteins associated with steroidogenesis, including mtTFA, COX4, and StAR. Furthermore, the levels of antioxidants, such as SOD2 and GPx4, which help prevent oxidative stress, increased in response to resveratrol supplementation. The study also found that resveratrol supplementation corrected the autophagy deficit associated with the HFD, as evidenced by the detection of autophagosomes and changes in the expression of autophagy‐related proteins (LC3II/I, Beclin1 and Atg7) (Wang et al. 2022). Additional research using autophagy inhibitors revealed that in H_2_O_2_‐challenged TM3 cells, autophagy inhibition partially nullified the protective effects of resveratrol on mitochondrial activity and steroidogenesis. However, in Leydig cells, the co‐administration of resveratrol and the autophagy activator rapamycin did not enhance defense against oxidative damage (Wang et al. 2022).

Testosterone synthesis is one of the steroid hormones that might be adversely affected by an HFD (Pinto‐Fochi et al. 2016). Research has demonstrated that supplementing with resveratrol can lessen the reduction in steroidogenesis brought on by an HFD (Wang et al. 2022). By activating AMPK, which stimulates the AMPK/mTOR (mammalian target of rapamycin) pathway, resveratrol affects steroidogenesis and mitochondrial function at least partially (Martin‐Hidalgo et al. 2018; Liu, Sun, and Li 2018). The control of autophagy and cellular energy balance depends heavily on this system. A sensor of cellular energy called AMPK controls several metabolic functions. The activation of AMPK by resveratrol can increase mitochondrial function, stimulate the production of steroidogenic enzymes, and encourage their biogenesis (Martin‐Hidalgo et al. 2018; Liu, Sun, and Li 2018). Leydig cells, which are responsible for testosterone production, use autophagy to facilitate the trafficking of cholesterol, a chemical that serves as a building block for the manufacture of testosterone. Autophagic vesicles move cholesterol from the outside to the inner mitochondrial membrane, where it is used to produce testosterone (Yi and Tang 1995). This cholesterol transport pathway can be interfered with by autophagy malfunction, which lowers testosterone production (Gao et al. 2018). Leydig cells' control over oxidative stress has also been linked to autophagy. Mitochondria primarily generate ROS, the key location of testosterone production in Leydig cells (Li et al. 2021; Filomeni et al. 2015). An overabundance of ROS can hinder steroidogenesis and lead to oxidative damage to mitochondria. Proper steroidogenesis is promoted by autophagy because it reduces oxidative stress and eliminates damaged mitochondria. Research has demonstrated that autophagy abnormalities in Leydig cells can cause lower testosterone levels and poor steroidogenesis.

Disorders affecting autophagy, such as obesity brought on by an HFD, can cause Leydig cell malfunction (Namkoong et al. 2018). On the other hand, increasing autophagy activity has been demonstrated to enhance steroidogenesis and shield Leydig cells from oxidative stress. Common in NASH, muscle atrophy is linked to the illness's progression and a higher chance of developing severe hepatic fibrosis (Koo et al. 2017). Liu et al. (Liu et al. 2020) showed that resveratrol treatment improved grip strength, muscle mass, and exercise performance in NASH mice. In the muscles of NASH mice, the researchers discovered a negative association between muscle SIRT1 activity and oxidative stress levels. Resveratrol positively affected oxidative stress suppression, antioxidant upregulation, protein degradation inhibition, autophagy activation, apoptosis suppression, and lipolytic gene upregulation. It decreased fatty infiltration in the limb muscles of NASH mice. Resveratrol prevented oxidative stress, lipid accumulation, autophagy malfunction, and palmitate acid‐induced apoptosis in in vitro muscle cell studies (Liu et al. 2020). The use of a specific SIRT1 inhibitor reversed these effects. In NASH mice, resveratrol‐induced SIRT1 activation has been shown to control autophagy, promoting removal of damaged cellular components and preserving muscle homeostasis (Liu et al. 2020). NAFLD and its more severe variant, NASH, can be treated therapeutically by increasing skeletal muscle mass (Hallsworth et al. 2011; Bacchi et al. 2013).

### 7.4 Autophagy and Hepatocellular Cancer

Autophagy, a vital process for cellular homeostasis, plays a paradoxical role in HCC, the most common type of primary liver cancer (Huang et al. 2018). Autophagy is increasingly recognized as a double‐edged sword, acting as a tumor suppressor and promoter depending on the cellular context and disease stage (Huang et al. 2018). In the early phases of liver carcinogenesis, autophagy helps prevent malignant transformation by eliminating damaged organelles and misfolded proteins, thereby reducing oxidative stress, preventing DNA damage, and maintaining genomic integrity. These actions protect hepatocytes from oncogenic mutations and chronic inflammation, ultimately hindering the initiation of tumor development (Huang et al. 2018).

However, in established HCC, autophagy supports tumor cell survival under adverse conditions such as hypoxia, nutrient deprivation, and therapeutic stress. It facilitates recycling intracellular components to sustain energy metabolism and anabolic processes required for rapid cell proliferation. Additionally, autophagy enhances epithelial‐mesenchymal transition (EMT), increasing tumor invasiveness and metastasis. It also plays a crucial role in maintaining cancer stem cells, which are associated with resistance to chemotherapy and radiotherapy. Thus, while autophagy initially acts as a barrier to tumorigenesis, it later becomes a survival mechanism that enables tumor progression and therapeutic resistance.

The regulation of autophagy in HCC involves a complex network of signaling pathways, each contributing uniquely to the disease process. One of the most critical regulatory axes is the PI3K/AKT/mTOR pathway (Yang et al. 2019). Activation of AKT inhibits the TSC1/TSC2 complex, leading to the activation of mTORC1, a well‐known suppressor of autophagy. mTORC1 inhibits the initiation of autophagy by blocking the ULK1 complex (Dossou and Basu 2019). Nutrient availability and growth factor signaling influence this pathway, while the tumor suppressor PTEN can negatively regulate PI3K signaling, thus promoting autophagy when functional.

Another key pathway is the AMPK/mTOR axis. AMPK, an energy sensor, becomes activated under low‐energy conditions and inhibits mTORC1 or directly activates ULK1, thereby initiating autophagy (Alers et al. 2012). This mechanism is especially relevant in the tumor microenvironment, where cancer cells often face metabolic stress. The IGF‐EGFR‐MAPK pathway also contributes to autophagy regulation in HCC (Liu et al. 2017; Zheng et al. 2025). Growth factors like IGF and EGF activate downstream cascades—including PI3K/AKT, Ras/Raf/ERK, and JNK/c‐Jun—which are involved in cell survival and autophagy induction (Zheng et al. 2025).

The Wnt/β‐catenin pathway plays a significant role in autophagy and tumor progression (Su et al. 2016; Lorzadeh et al. 2021; Ma et al. 2024). In the absence of Wnt signals, β‐catenin is degraded by the proteasome. However, when Wnt is present, β‐catenin accumulates and translocates to the nucleus, activating target genes involved in proliferation and autophagy regulation. Dysregulation of this pathway is frequently observed in HCC and contributes to cancer stemness and resistance mechanisms (Ma et al. 2024).

Autophagy is also modulated by the tumor suppressor p53, which exhibits dual effects depending on cellular localization (Rahman et al. 2022; Verma et al. 2021; Pang and Liu 2018; Yazdani et al. 2019). In the nucleus, p53 promotes autophagy by activating the AMPK and inhibiting the PI3K/AKT/mTOR pathways. Conversely, cytoplasmic p53 can repress autophagy, highlighting the complexity of its role. The NF‐κB pathway further adds to this regulatory complexity. NF‐κB can induce autophagy by upregulating Beclin‐1 and other autophagy‐related genes, yet it may also suppress autophagy via upregulation of anti‐apoptotic proteins such as Bcl‐2, Bcl‐xL, and BNIP3 (Trocoli and Djavaheri‐Mergny 2011; Verzella et al. 2020; Decuypere et al. 2012).

These intricate signaling networks underline the importance of understanding autophagy's role in HCC. Modulating autophagy presents a promising therapeutic approach but requires careful consideration of the tumor's stage and molecular profile. In early‐stage HCC or precancerous lesions, promoting autophagy may help eliminate potentially malignant cells and improve immune responses. In contrast, inhibiting autophagy in advanced HCC may sensitize tumors to chemotherapy, radiotherapy, and targeted therapies such as sorafenib (Xie et al. 2023).

### 7.5 Autophagy Induction by Resveratrol in Hepatocellular Carcinoma

Natural compounds such as resveratrol have shown promise in modulating autophagy through several pathways. Preclinical studies indicate that resveratrol can induce autophagic cell death and enhance the efficacy of existing chemotherapeutic agents. Numerous studies have demonstrated that resveratrol exerts its antitumor effects by modulating autophagy, apoptosis, and cellular signaling pathways (see Table 4). Zhang et al. (2018) revealed that resveratrol suppresses proliferation, viability, and metastasis of HCC cells by upregulating the tumor suppressor gene p53 and downregulating the PI3K/Akt signaling pathway, leading to autophagy induction. These molecular changes promote the degradation of oncogenic cellular components, thereby limiting tumor progression.

**TABLE 4:** Resveratrol‐Mediated Modulation of Autophagy in Hepatocellular Carcinoma.

Author (Year)	Dosage of resveratrol	Duration	Experimental model	Mechanistic role	Autophagy or molecular marker changes	Key findings
Zhang et al. (2018)	0, 25, 50, 100 μM	24, 48, 72 h	Human HCC cell lines (HepG2, Huh‐7)	↑ p53 expression ↓ p‐Akt/Akt ratio→ Induces autophagy via p53 activation & PI3K/Akt inhibition	↑ Beclin1 ↑ LC3‐II/I ratio ↑ LC3+ puncta formation ↓ p62 expression	Resveratrol inhibited HCC cell proliferation, invasion, and migration via autophagy induction; effects were reversed by autophagy, p53, and Akt modulation.
Song et al. (2021)	6.25 μmol/L	Not specified	Human liver cancer cell lines (HepG2, Huh7)	↑ miR‐186‐5p expression→ Inhibition of EMT and metastasis‐related pathways	↑ E‐cadherin (epithelial marker) ↓ Vimentin, Twist1 (mesenchymal markers) ↑ miR‐186‐5p expression	Resveratrol inhibited migration, invasion, and EMT via upregulation of miR‐186‐5p. Blocking miR‐186‐5p reversed these effects.
Tomas‐Hernández et al. (2018)	RSV: 50 μM QCT: 50 μM	Not explicitly stated	Human liver cancer cells (HepG2)	RSV alone: ↑ autophagy RSV + QCT: ↓ QCT‐induced autophagy ↑ apoptosis via AMPK modulation, LMP, Zn^2+^ dynamics	↓ HO‐1 expression ↑ AMPK phosphorylation ↑ Lysosomal membrane permeabilization (LMP) Zn^2+^ efflux linked to apoptosis	RSV counters QCT‐induced autophagy and sensitizes HepG2 cells to apoptosis through LMP and AMPK activation mechanisms.
Tong et al. (2024)	Not explicitly stated	Not clearly stated	Human liver cancer cells (Huh7)	↓ Exosome secretion via ↓ Rab27a Exosomes mediate ↓ β‐catenin nuclear translocation and ↓ autophagy via lncRNA SNHG29	↓ Rab27a ↓ nuclear β‐catenin ↓ autophagy activity lncRNA SNHG29 mediates these effects	Resveratrol inhibits HCC progression by suppressing exosome secretion and modifying exosome cargo that limits EMT and proliferation.
Feng et al. (2025)	Low, medium, high doses	In vitro & in vivo (unspecified exact days)	HepG2 cells, HCC xenograft mice	Induces mitophagy via ↓ MALAT1 → ↑ miR‐143‐3p → ↓ RRM2 Enhances apoptosis, reduces proliferation	↑ Mitophagy markers (assessed by TEM, immunofluorescence, WB) ↓ RRM2 expression ↑ mitochondrial dysfunction (ROS, ↓ membrane potential)	Resveratrol inhibits HCC progression by activating mitophagy through the MALAT1/miR‐143‐3p/RRM2 pathway, highlighting a novel antitumor mechanism.

In addition to p53‐related signaling, resveratrol has been shown to inhibit the EMT, a critical process in cancer metastasis. This inhibition is mediated through the upregulation of miR‐186–5p, a microRNA that regulates autophagy and apoptosis in HCC (Song et al. 2021). Although the exact mechanism of resveratrol's influence on the miR‐186–5p pathway remains underexplored, its impact on EMT suppression highlights its therapeutic promise. Furthermore, resveratrol treatment results in upregulating autophagy markers Beclin1 and LC3‐II and downregulating p62, a protein accumulating when the autophagic flux is impaired. These dose‐dependent changes reinforce the compound's role in stimulating effective autophagic degradation.

Interestingly, when combined with quercetin (QCT)—another bioactive flavonoid known to induce autophagy—resveratrol demonstrates a dual role (Tomas‐Hernández et al. 2018). While both compounds independently enhance autophagy, their combination unexpectedly reduces QCT‐induced autophagy and activates pro‐apoptotic signaling in HepG2 cells (Tomas‐Hernández et al. 2018). Mechanistic studies suggest that this interaction may depend on the cellular energetic state, with AMPK phosphorylation, heme oxygenase‐1 (HO‐1) downregulation, and lysosomal membrane permeabilization (LMP) identified as key mediators of this effect (Tomas‐Hernández et al. 2018). These findings imply that resveratrol may sensitize HCC cells to other treatments by modulating intracellular stress responses.

Moreover, resveratrol affects exosome secretion and content modulation in HCC (Tong et al. 2024). It downregulates Rab27a, a protein essential for exosome release, inhibiting cell proliferation, migration, and EMT. Resveratrol‐induced exosomes suppress β‐catenin nuclear translocation and attenuate autophagy, potentially mediated by lncRNA SNHG29 (Tong et al. 2024). This further suggests that resveratrol's anti‐HCC effects are multifaceted and extend beyond classical intracellular pathways.

Additional studies reinforce resveratrol's dose‐ and time‐dependent antitumor effects in HCC, including inhibition of cell viability, proliferation, invasion, and migration. These effects are strongly linked to autophagy, as demonstrated by elevated Beclin1 levels, increased LC3‐II/I ratio, and decreased p62 expression following resveratrol treatment. Moreover, the autophagy inhibitor 3‐methyladenine (3‐MA) counteracted resveratrol's tumor‐suppressive actions, indicating that autophagy activation is critical to resveratrol's efficacy (Zhang et al. 2018). The role of p53 activation and reduced p‐Akt/Akt ratio was also confirmed as upstream events leading to autophagic induction and inhibition of HCC progression (Zhang et al. 2018).

Recent research has expanded the scope of resveratrol's action by demonstrating its ability to induce mitophagy, a selective autophagy targeting damaged mitochondria (Feng et al. 2025). Using both in vitro and in vivo models, resveratrol impairs mitochondrial function while promoting mitophagy in HCC cells and tumor‐bearing mice. This process is mediated through the MALAT1/miR‐143‐3p/RRM2 axis, wherein resveratrol downregulates the oncogenic lncRNA MALAT1, upregulates miR‐143‐3p, and inhibits the expression of RRM2, a known promoter of tumor progression (Feng et al. 2025). Disruption of this axis potentiated the antitumor effects of resveratrol and confirmed its role in mitochondrial quality control via mitophagy.

These findings position resveratrol as a potent antitumor agent in HCC through activation of autophagy and mitophagy, modulation of tumor‐related microRNAs and lncRNAs, and inhibition of critical oncogenic signaling pathways. Its ability to act on multiple cellular targets underscores its potential in combination therapies to sensitize HCC cells to autophagy‐based interventions. Future clinical investigations are warranted to validate these preclinical results and explore resveratrol's full therapeutic utility in liver cancer management.

## 8 Conclusions

Resveratrol exerts multifaceted protective effects against liver pathologies such as NAFLD and HCC, primarily through the modulation of apoptosis, autophagy, oxidative stress, and inflammation. By targeting key regulatory pathways, including PI3K/AKT/mTOR and SIRT1, resveratrol promotes autophagy and antioxidant defense while attenuating pro‐apoptotic and pro‐inflammatory signaling. The therapeutic efficacy of resveratrol is context‐dependent: it enhances hepatocyte survival and metabolic balance in NAFLD, yet may inhibit apoptosis in advanced HCC, which could support tumor progression. Therefore, while resveratrol holds promise as a natural therapeutic agent, its application in liver disease treatment should consider disease stage and molecular context to maximize benefits and avoid unintended consequences.

## Author Contributions

**Ziyao Wan:** conceptualization (equal), writing – original draft (equal), writing – review and editing (equal). **Jamal Hallajzadeh:** conceptualization (equal), writing – original draft (equal), writing – review and editing (equal).

## Ethics Statement

The authors have nothing to report.

## Consent

The authors have nothing to report.

## Conflicts of Interest

The authors declare no conflicts of interest.

## Data Availability

The authors have nothing to report.
